# Epidemiology of Idiopathic Central Serous Chorioretinopathy in Taiwan, 2001–2006: A Population-based Study

**DOI:** 10.1371/journal.pone.0066858

**Published:** 2013-06-24

**Authors:** Der-Chong Tsai, Shih-Jen Chen, Chin-Chou Huang, Pesus Chou, Chia-Min Chung, Po-Hsun Huang, Shing-Jong Lin, Jaw-Wen Chen, Tseng-Ji Chen, Hsin-Bang Leu, Wan-Leong Chan

**Affiliations:** 1 Institute of Public Health and Community Medicine Research Center, National Yang-Ming University, Taipei, Taiwan, R.O.C; 2 Department of Ophthalmology, National Yang-Ming University Hospital, Yilan, Taiwan, R.O.C; 3 Department of Ophthalmology, Taipei Veterans General Hospital, Taipei, Taiwan, R.O.C; 4 Institute of Pharmacology, National Yang-Ming University, Taipei, Taiwan, R.O.C; 5 Cardiovascular Research Center, School of Medicine, National Yang-Ming University, Taipei, Taiwan, R.O.C; 6 Division of Cardiology, Department of Medicine, Taipei Veterans General Hospital, Taipei, Taiwan, R.O.C; 7 Department of Medical Research and Education, Taipei Veterans General Hospital, Taipei, Taiwan, R.O.C; 8 Institute of Biomedical Sciences, Academia Sinica, Taipei, Taiwan, R.O.C; 9 Institute of Clinical Medicine, National Yang-Ming University, Taipei, Taiwan, R.O.C; 10 Healthcare and Management Center, Taipei Veterans General Hospital, Taipei, Taiwan, R.O.C; 11 Department of Family Medicine, Taipei Veterans General Hospital, Taipei, Taiwan, R.O.C; 12 Institute of Hospital and Health Care Administration, National Yang-Ming University, Taipei, Taiwan, R.O.C; Zhongshan Ophthalmic Center, China

## Abstract

**Objectives:**

The epidemiology of idiopathic central serous chorioretinopathy (CSCR) is not well understood in an Asian population. The present study aimed to investigate the incidence and risk factors for corticosteroid-unrelated CSCR using Taiwan’s National Health Insurance Research Database.

**Methods and Results:**

From 2001 to 2006, a total of 786 patients (500 [63.6%] males) who were newly diagnosed with CSCR, aged from 20 to 64 years and had no history of corticosteroid prescription were identified as incident cases of idiopathic CSCR. 3606 age-, gender-, and enrollment time-matched subjects were randomly selected as the control group. The mean annual incidence was 0.21‰ (0.27‰ for males, and 0.15‰ for females; *P*<0.001), with a male/female ratio of 1.74. The peak incidence was in the 35- to 39-year-old age group (0.30‰), followed by the 40- to 44-year-old age group (0.26‰). Males had a significantly higher mean annual incidence than female only in the middle age groups. Conditional logistic regression was used to estimate the odds ratios (ORs) for potential risk factors of idiopathic CSCR. Only exposure to anti-anxiety drugs (OR, 1.63; 95% confidence interval, 1.09–2.44) was found to be independently associated with idiopathic CSCR among males. No risk factors of idiopathic CSCR were found for females.

**Conclusions:**

This study provides the nationwide, population-based data on the incidence of idiopathic CSCR in adult Asians, and suggests that exposure to anti-anxiety drugs is an independent risk factor for idiopathic CSCR among males.

## Introduction

Central serous chorioretinopathy (CSCR) is a common maculopathy mainly affecting young and middle-aged adults and occurring more frequently in men than in women [1]–[3]. The acute manifestation of CSCR is characterized by fluid accumulation in the subretinal space and/or retinal pigment epithelial (RPE) detachment which usually resolves spontaneously within a few months with good visual recovery [1]–[3]. Previous case series and case-control studies have found that CSCR is associated with psychological stress, endogenous hypercortisolism, and corticosteroid mediation [4]–[14]. Among non-surgical retinopathy, CSCR has been reported as the fourth most common after age-related macular degeneration (AMD), diabetic retinopathy, and branched retinal vein occlusion [1]. Racial variations in the prevalence of CSCR have been reported, with high rates in Caucasians and Hispanics and higher rates in Asians, but lower rates in African Americans [1], [3], [15]–[17]. However, there is limited information on the incidence of CSCR. The only population-based study, conducted in Olmsted County, Minnesota, found a mean annual incidence of 5.8 per 100,000 in a predominantly Caucasian population [18].

Although the exact mechanism remains unclear, exogenous corticosteroid use may trigger or exacerbate CSCR and it has been suggested that this maculopathy should be added to the list of the ophthalmic complications of corticosteroids [10]. Therefore, CSCR unrelated to corticosteroid treatment is considered “idiopathic” [11], [14]. In the published literature, most cases of CSCR have been reported to be idiopathic [8]–[11]. In addition to corticosteroid use, a number of risk factors for CSCR, such as hypertension, psychopharmacologic medication use and pregnancy, have been reported. However, most of these systemic associations have been inconsistent between the hospital-based and population-based case-control studies [8], [9], [18].

As there is very limited knowledge of the incidence of idiopathic CSCR in Asian populations, and as the associated risk factors remain undetermined, we conducted this nationwide study with a retrospective case-control study design to investigate the epidemiology of idiopathic CSCR using the Taiwan National Health Insurance database.

## Methods

### Database

The National Health Insurance (NHI) program in Taiwan, a mandatory, single-payer social health insurance system, was initiated in 1995 and currently has more than 22 million enrollees, representing nearly 99% of the entire population of Taiwan. The National Health Insurance Research Database (NHIRD), containing all original claims data from the NHI program, is published annually by the Taiwan National Health Research Institute (NHRI) in an electronically encrypted form. It is one of the largest and most comprehensive nationwide population-based databases in the world and has been used extensively in epidemiological studies [19]. A sample cohort dataset composed of one million randomly sampled beneficiaries covered by the NHI program in the year 2000 was created by the NHRI for the research purposes. This random sample has been confirmed by the NHRI to be representative of the Taiwanese population under the NHI program, and all of their claims data are available from 1996 onwards. Therefore, we analyzed this representative cohort dataset to explore the incidence and systemic risk factors for idiopathic CSCR in this study. The Institutional Review Board of National Yang-Ming University Hospital approved the study and waived the requirement of informed consent due to analyzing datasets in a database, which is devoid of identifiable personal information.

### Study Sample

From the aforementioned cohort dataset, subjects newly diagnosed as cases of CSCR (*International Classification of Diseases, Ninth Revision, Clinical Modification* [ICD-9-CM] code 362.41), with no history of exogenous corticosteroid use, and aged from 20 to 64 years between January 1, 2001 and December 31, 2006 were identified as incident patients with idiopathic CSCR. CSCR is defined as a localized serous detachment of the neurosensory retina in the macula not associated with subretinal blood or lipid exudate. Those who had been diagnosed with CSCR before 2001 were not included to increase the likelihood of excluding preexisting chronic or recurrent CSCR patients. Additionally, to help rule out other causes for RPE leaks in the differential diagnosis of CSCR, we also excluded those who had ever had any of the following diagnoses before enrollment and within the first year after enrollment: degenerative myopia (ICD-9-CM code 360.21), hemorrhagic RPE detachment (362.43), exudative AMD (362.52), macular hole (362.54), hereditary retinal dystrophies (362.7x), focal chorioretinitis (363.0x), disseminated chorioretinitis (363.1x), Harada’s disease (363.22), angioid streak (363.43), or malignant neoplasm of the choroid (190.6).

To determine the risk factors for idiopathic CSCR, enrollees who had never been diagnosed with CSCR in the sample cohort dataset were selected as the control group using the stratified random sampling method. Matching characteristics included age, gender, and the time of enrollment. To reduce the likelihood of misclassifying the patients with undetected CSCR in the control group, we excluded those who had never sought ambulatory or inpatient care provided by ophthalmologists during an 8-year period from 2000 to 2007. Furthermore, potential controls were also excluded if they had ever received corticosteroid treatment or been diagnosed as having degenerative myopia, hemorrhagic detachment of the RPE, exudative AMD, macular hole, hereditary retinal dystrophies, focal chorioretinitis, disseminated chorioretinitis, Harada’s disease, angioid streak, or malignant neoplasm of the choroid during the study period.

### Variables of Interest

The preexisting medical conditions of interest included hypertension (ICD-9-CM codes 401.xx –405.xx), diabetes mellitus (250.xx), hyperlipidemia (272.xx), chronic renal disease (580.xx –587.xx), sleep apnea (780.51, 780.53, 780.57), gout (274.xx), malignancy (140.0–199.1), allergic respiratory diseases (including asthma [493.xx], allergic rhinitis [477.x], or sinusitis [473.x]), autoimmnue diseases (including Graves’ disease [242.xx], sarcoidosis [135], psoriasis [696.x], connective tissue disease [710.x], Reiter’s syndrome [711.1x], rheumatoid arthritis [714.0], ankylosing spondylitis [720.x], thrombocytopenic purpura [287.xx], ulcerative colitis [556.x], or Crohn’s disease [555.x]), pregnancy (V22.x, V23.x, V27.x, V28.x), and anti-anxiety medication usage. Prescription medications were identified through National Drug Codes on outpatient and inpatient prescription drug claims. Exposure to anti-anxiety drugs for more than one month was examined among all subjects within a one-year period before enrollment. All these preexisting medical conditions were considered as potential risk factors and compared between the study group and the control group.

### Statistical Analysis

Microsoft SQL Server 2008 was used for data management and computing. Statistical analysis was performed using SPSS software (version 17.0, SPSS Inc., Chicago, Illinois, USA). All data were expressed as mean ± standard deviation (SD) or percentage. Annual incidence and sex-age-specific mean annual incidence rates from 2001 to 2006 were calculated. Age was categorized into one of nine groups: 20 to 24, 25 to 29, 30 to 34, 35 to 39, 40 to 44, 45 to 49, 50 to 54, 55 to 59, and 60 to 64 years old. Annual incidence for idiopathic CSCR was calculated as the number of incident cases per 1,000 person-years of the given year. The sampled subjects who were not diagnosed with CSCR at the end of a given year contributed one person-year to the denominator of the incidence rate. Those who were newly diagnosed with idiopathic CSCR during a given year contributed one-half person-year to the denominator of the incidence rate. For comparisons of mean annual incidences and medical histories between genders, Pearson’s χ^2^ tests were applied. The independent Student’s t-tests were used to examine differences between the study group and the control group of continuous data. In terms of sociodemographic characteristics and the prevalence of medical conditions of interest, differences between the two groups were determined by Pearson’s χ^2^ tests. Multivariate conditional logistic regression models (controlled for all variables listed above) were constructed by gender to estimate the odds ratios (ORs) for potential risk factors for idiopathic CSCR. Statistical significance was inferred at a two-sided p value of less than 0.05.

## Results

From 2001 to 2006, a total of 786 incident cases with idiopathic CSCR were identified from the sampling cohort dataset and eligible for inclusion criteria in the case group. Of these subjects, 500 (63.6%) were male and 286 (36.4%) were female. The mean age at diagnosis was 39.3±10.5 years (median age, 39 years).

Table 1 lists the annual incidence of idiopathic CSCR from 2001 to 2006 among the National Health Insurance enrollees aged from 20 to 64 years. The annual incidences were relative stable and ranged from 0.18‰ in 2001 and 2004 to 0.24‰ in 2006. The mean annual incidence was 0.21‰. Table 2 shows the age- and sex- specific mean annual incidence rates (2001–2006) of the study subjects. The mean annual incidence was 0.27‰ for males and 0.15‰ for females (P<0.001), with a male/female ratio of 1.74. Males had a significantly higher mean annual incidence of idiopathic CSCR than females in each age group from age 35 to 54 years (all P<0.001). The peak mean annual incidence was in the 35- to 39-year-old age group (0.30‰), followed by the 40- to 44-year-old age group (0.26‰). Table 3 shows the comparisons of preexisting medical conditions between the female and male patients by age. There were no significant differences between genders in the younger and older groups. However, male patients were more likely to have hypertension (11.0% versus 3.4%; P = 0.032) than female patients in the middle age group.

**Table 1:** Annual Incidence of Idiopathic Central Serous Chorioretinopathy, 2001–2006.

Year	Incident Cases	Person-Years	Annual Incidence (‰)
2001	113	614,796	0.18
2002	119	620,004	0.19
2003	138	625,904	0.22
2004	111	631,561	0.18
2005	148	635,947	0.23
2006	157	639,860	0.24
Average	131	628,012	0.21

**Table 2:** Age- and Sex-Specific Annual Incidence of Idiopathic Central Serous Chorioretinopathy, 2001–2006.

	Mean Annual Incidence (‰)			
Age, years	Total	Male	Female	*p* value
**20–24**	0.12	0.10	0.14	0.223
**25–29**	0.21	0.18	0.23	0.254
**30–34**	0.20	0.20	0.19	0.739
**35–39**	0.30	0.44	0.17	<0.001
**40–44**	0.26	0.40	0.11	<0.001
**45–49**	0.20	0.33	0.08	<0.001
**50–54**	0.22	0.30	0.13	<0.001
**55–59**	0.21	0.21	0.20	0.869
**60–64**	0.12	0.13	0.11	0.587
**Total**	0.21	0.27	0.15	<0.001

**Table 3:** Comparisons between Female and Male Patients with Idiopathic Central Serous Chorioretinopathy by Age.

	**20 to 34 years old**			**35 to 49 years old**			**50 to 64 years old**		
	**Female**	**Male**		**Female**	**Male**		**Female**	**Male**	
**Medical history, n (%)**	**(n = 139)**	**(n = 123)**	***p*** ** value**	**(n = 88)**	**(n = 283)**	***p*** ** value**	**(n = 59)**	**(n = 94)**	***p*** ** value**
Hypertension	1 (0.7)	2 (1.6)	0.602	3 (3.4)	31 (11.0)	**0.032**	15 (25.4)	26 (27.7)	0.761
Diabetes mellitus	1 (0.7)	0	1.000	2 (2.3)	9 (3.2)	1.000	7 (11.9)	10 (10.6)	0.814
Hyperlipidemia	0	0	-	4 (4.5)	26 (9.2)	0.163	13 (22.0)	13 (13.8)	0.188
Chronic kidney disease	2 (1.4)	1 (0.8)	1.000	2 (2.3)	9 (3.2)	1.000	1 (1.7)	4 (4.3)	0.649
Sleep apnea	0	1 (0.8)	0.469	0	1 (0.4)	1.000	1 (1.7)	0	0.386
Gout	1 (0.7)	6 (4.9)	0.054	2 (2.3)	22 (7.8)	0.067	5 (8.5)	6 (6.4)	0.750
Malignancy	2 (1.4)	0	0.500	0	3 (1.1)	1.000	1 (1.7)	4 (4.3)	0.649
Autoimmune disorders*	4 (2.9)	6 (4.9)	0.523	7 (8)	13 (4.6)	0.277	4 (6.8)	2 (2.1)	0.206
Allergic respiratory disorders^†^	16 (11.5)	15 (12.2)	0.864	9 (10.2)	30 (10.6)	0.921	13 (22.0)	14 (14.9)	0.259
Exposure to anti-anxietydrugs	0	2 (1.6)	0.219	3 (3.4)	17 (6.0)	0.429	9 (15.3)	9 (9.6)	0.289

*P* value less than 0.05 in bold.

To investigate the systemic association of idiopathic CSCR, a total of 3606 subjects (mean age 39.3±10.6 years; 2294 [63.6%] males), matched for age, gender, and enrollment time, were recruited as the control group. Table 4 and Table 5 present the distributions of sociodemographic characteristics and medical histories for the cases and the controls by gender. Male patients with idiopathic CSCR were more likely to have hypertension (11.8% versus 8.7%; P = 0.031) and exposure to anti-anxiety drugs (5.6% versus 3.0%; P = 0.004) compared to male controls. However, there were no significant differences in the distribution of sociodemographic characteristics and medical conditions of interest between female cases and female controls. Table 6 and Table 7 show the results of conditional logistic regression analysis by gender. After adjusting for monthly income, place of residence, medical history including hypertension, diabetes mellitus, hyperlipidemia, chronic renal disease, sleep apnea, gout, malignancy, autoimmune disorders, allergic respiratory disorders, and exposure to anti-anxiety drugs, only exposure to anti-anxiety drugs (OR, 1.63; 95% confidence interval [CI], 1.09–2.44; P = 0.019) was independently associated with a diagnosis of idiopathic CSCR among males. No risk factors of idiopathic CSCR were found for females.

**Table 4:** Characteristics of Male Cases and Controls.

	Male cases	Male controls	
	(n = 500)	(n = 2294)	*p* value
Age, mean years (SD)	40.5 (9.5)	40.4 (9.8)	0.731
Monthly income, n (%)			0.536
<NT$15,000	104 (20.8)	506 (22.1)	
NT$15,000–30,000	203 (40.6)	952 (41.5)	
NT$30,001–50,000	115 (23.0)	534 (23.3)	
>NT$50,000	78 (15.6)	302 (13.2)	
Place of residence, n (%)			0.338
Northern	232 (46.4)	1027 (44.8)	
Central	128 (25.6)	631 (27.5)	
Southern	127 (25.4)	600 (26.2)	
Eastern	13 (2.6)	36 (1.6)	
Medical history, n (%)			
Hypertension	59 (11.8)	200 (8.7)	**0.031**
Diabetes mellitus	19 (3.8)	102 (4.4)	0.520
Hyperlipidemia	39 (7.8)	152 (6.6)	0.346
Chronic kidney disease	14 (2.8)	36 (1.6)	0.060
Sleep apnea	2 (0.4)	5 (0.2)	0.616
Gout	34 (6.8)	178 (7.8)	0.463
Malignancy	7 (1.4)	28 (1.2)	0.744
Autoimmune disorders*	21 (4.2)	93 (4.1)	0.881
Allergic respiratory disorders†	59 (11.8)	225 (9.8)	0.182
Exposure to anti-anxiety drugs	28 (5.6)	69 (3.0)	**0.004**

SD, standard deviation; NT$, New Taiwan Dollar.

* Including Graves’ disease, sarcoidosis, psoriasis, connective tissue disease, Reiter’s syndrome, rheumatoid arthritis, ankylosing spondylitis, thrombocytopenic purpura, ulcerative colitis, and Crohn’s disease.

† Including asthma, allergic rhinitis, and sinusitis.

*P* value less than 0.05 in bold.

**Table 5:** Characteristics of Female Cases and Controls.

	Female cases	Female controls	
	(n = 286)	(n = 1312)	*p* value
Age, mean years (SD)	37.2 (11.8)	37.4 (11.5)	0.793
Monthly income, n (%)			0.884
<NT$15,000	87 (30.4)	431 (32.9)	
NT$15,000–30,000	114 (39.9)	508 (38.7)	
NT$30,001–50,000	69 (24.1)	301 (22.9)	
>NT$50,000	16 (5.6)	72 (5.5)	
Place of residence, n (%)			0.136
Northern	84 (29.4)	370 (28.2)	
Central	157 (54.9)	782 (59.6)	
Southern	39 (13.6)	149 (11.4)	
Eastern	6 (2.1)	11 (0.8)	
Medical history, n (%)			
Hypertension	19 (6.6)	93 (7.1)	0.789
Diabetes mellitus	10 (3.5)	53 (4.0)	0.669
Hyperlipidemia	17 (5.9)	54 (4.1)	0.174
Chronic kidney disease	5 (1.7)	20 (1.5)	0.792
Sleep apnea	1 (0.3)	1 (0.1)	0.326
Gout	8 (2.8)	19 (1.4)	0.126
Malignancy	3 (1.0)	21 (1.6)	0.602
Autoimmune disorders*	15 (5.2)	78 (5.9)	0.647
Allergic respiratory disorders†	38 (13.3)	162 (12.3)	0.664
Exposure to anti-anxiety drugs	12 (4.2)	59 (4.5)	0.823
Pregnancy	10 (3.5)	65 (5.0)	0.291

SD, standard deviation; NT$, New Taiwan Dollar.

* Including Graves’ disease, sarcoidosis, psoriasis, connective tissue disease, Reiter’s syndrome, rheumatoid arthritis, ankylosing spondylitis, thrombocytopenic purpura, ulcerative colitis, and Crohn’s disease.

† Including asthma, allergic rhinitis, and sinusitis.

**Table 6:** Crude and Adjusted Odds Ratios for Idiopathic Central Serous Chorioretinopathy among Male Cases and Controls.

	Crude OR	(95% CI)	Adjusted OR	(95% CI)
Monthly income, n (%)				
<NT$15,000	Reference		Reference	
NT$15,000–30,000	1.02	(0.80–1.30)	1.04	(0.82–1.33)
NT$30,001–50,000	1.03	(0.78–1.35)	1.05	(0.80–1.39)
>NT$50,000	1.18	(0.87–1.60)	1.20	(0.88–1.64)
Place of residence, n (%)				
Northern	Reference		Reference	
Central	0.93	(0.74–1.15)	0.94	(0.76–1.18)
Southern	0.94	(0.76–1.17)	0.95	(0.76–1.18)
Eastern	1.45	(0.83–2.54)	1.49	(0.85–2.61)
Medical history, n (%)				
Hypertension	**1.34**	**(1.01–1.79)**	1.27	(0.93–1.74)
Diabetes mellitus	0.87	(0.54–1.38)	0.73	(0.44–1.21)
Hyperlipidemia	1.16	(0.83–1.62)	1.12	(0.76–1.64)
Chronic kidney disease	1.58	(0.93–2.70)	1.62	(0.95–2.79)
Sleep apnea	1.59	(0.40–6.41)	1.53	(0.38–6.19)
Gout	0.88	(0.62–1.25)	0.78	(0.54–1.13)
Malignancy	1.10	(0.52–2.33)	1.06	(0.50–2.25)
Autoimmune disorders*	1.05	(0.68–1.62)	0.99	(0.64–1.55)
Allergic respiratory disorders†	1.18	(0.90–1.55)	1.14	(0.87–1.51)
Exposure to anti-anxiety drugs	**1.64**	**(1.12–2.41)**	**1.63**	**(1.09–2.44)**

OR, odds ratio; CI, confidence interval; NT$, New Taiwan Dollar.

* Including Graves’ disease, sarcoidosis, psoriasis, connective tissue disease, Reiter’s syndrome, rheumatoid arthritis, ankylosing spondylitis, thrombocytopenic purpura, ulcerative colitis, and Crohn’s disease.

† Including asthma, allergic rhinitis, and sinusitis.

*P* value less than 0.05 in bold.

**Table 7:** Crude and Adjusted Odds Ratios for Idiopathic Central Serous Chorioretinopathy among Female Cases and Controls.

	Crude OR	(95% CI)	Adjusted OR	(95% CI)
Monthly income, n (%)				
<NT$15,000	Reference		Reference	
NT$15,000–30,000	1.12	(0.83–1.50)	1.11	(0.82–1.49)
NT$30,001–50,000	1.13	(0.81–1.58)	1.13	(0.81–1.58)
>NT$50,000	1.15	(0.66–1.99)	1.13	(0.64–1.97)
Place of residence, n (%)				
Northern	Reference		Reference	
Central	0.88	(0.68–1.16)	0.89	(0.68–1.17)
Southern	1.18	(0.80–1.74)	1.22	(0.82–1.81)
Eastern	1.72	(0.73–4.08)	1.79	(0.75–4.31)
Medical history, n (%)				
Hypertension	0.90	(0.54–1.51)	0.87	(0.50–1.50)
Diabetes mellitus	0.80	(0.41–1.54)	0.74	(0.37–1.47)
Hyperlipidemia	1.33	(0.79–2.25)	1.43	(0.81–2.50)
Chronic kidney disease	1.10	(0.45–2.71)	1.27	(0.52–3.14)
Sleep apnea	2.47	(0.34–17.81)	2.50	(0.33–18.80)
Gout	1.65	(0.81–3.39)	1.64	(0.78–3.43)
Malignancy	0.67	(0.21–2.11)	0.69	(0.22–2.17)
Autoimmune disorders*	0.87	(0.51–1.47)	0.85	(0.50–1.46)
Allergic respiratory disorders†	1.07	(0.76–1.51)	1.06	(0.75–1.50)
Exposure to anti-anxiety drugs	0.92	(0.51–1.67)	0.90	(0.49–1.68)
Pregnancy	0.75	(0.40–1.42)	0.76	(0.40–1.44)

OR, odds ratio; CI, confidence interval; NT$, New Taiwan Dollar.

* Including Graves’ disease, sarcoidosis, psoriasis, connective tissue disease, Reiter’s syndrome, rheumatoid arthritis, ankylosing spondylitis, thrombocytopenic purpura, ulcerative colitis, and Crohn’s disease.

† Including asthma, allergic rhinitis, and sinusitis.

## Discussion

To our knowledge, this study provides the first nationwide, population-based data on the incidence and risk factors for idiopathic CSCR in Taiwan by National Health Insurance Research Database. We found a mean annual incidence rate of 0.21‰ (0.27‰ for males and 0.15‰ for females) among those aged 20 to 64 years. The highest mean annual incidence was in the 35- to 39-year-old age group (0.30‰) and distributions of age-specific incidence were different between genders. In addition, only exposure to anti-anxiety drugs was significantly associated with idiopathic CSCR among males.

Similar to previous case-control studies [4], [8], [9], [11]–[13], [15], [18], [20], most of the 786 incident cases with idiopathic CSCR in our study were males (63.6%) and middle-aged (median age of 39 years). We found that the annual incidence of idiopathic CSCR (between 0.18‰ and 0.24‰) was relatively stable in Taiwan during the study period, with no obvious trend of decrease or increase. Kitzmann et al. recognized 74 angiographic-proven CSCR patients (including 6 [8%] cases under corticosteroid use) and reported that the mean annual incidence of CSCR was 5.8 per 100 000 in Olmsted County, Minnesota [18]. The incidence rate in an Asian population of our study was therefore approximately 4 times higher than that in a predominantly Caucasian population. The differences in ethnicity and environment between the study populations as well as the differences in methodology between the studies might explain the divergence of the incidence.

By reviewing the medical records between 1980 and 2002 in Olmsted County, Kitzmann et al. estimated the CSCR incidence with the inclusion criteria of a fluorescein angiogram [18]. Nevertheless, the incidence of CSCR could be underestimated with angiographic confirmation as a required inclusion criterion, because not all typical cases with acute exudative macular manifestation receive fluorescein angiography for diagnostic purpose. However, even including 9 cases without imaging studies, the CSCR incidence in Olmsted County was 7.19 per 100000 [18], which was still much lower than that in Taiwan. This finding is compatible with previous literatures reporting that CSCR is more common in the Asian populations than in the Caucasian populations [3],[21].

The mean annual incidence rate of idiopathic CSCR among males (0.27‰) was about 1.74-fold higher than that among females (0.15‰) in our study, and the male/female ratio is lower than the reported ratio of 5.85 in a previous population-based study [18]. Consistent with published literature, CSCR occurred most frequently in the middle-aged males, with a peak incidence at an age between 35 and 39 years (0.44‰) in our study. However, the mean annual incidence rates of females were relatively stable across all age groups when compared to those of males, and the typical pattern of middle-aged predominance shown in men was absent in women. The available data on CSCR in women is limited. Quillen et al reviewed the medical charts of 51 women with CSCR, and found that the clinical features were similar between genders [14]. In our study, there was no significant difference in the distribution of preexisting medical conditions between the female and male patients with idiopathic CSCR, except that males were more likely to have hypertension than females in the middle age group (from 35 to 50 years). This difference in hypertension prevalence could not account for the difference in idiopathic CSCR incidence between genders in the middle age group, as the prevalence of hypertension is not proportioned to the annual incidence of idiopathic CSCR. In addition, hypertension was not significantly related to idiopathic CSCR development in our study. Further investigations are warranted to explore the difference in incidence between genders.

In the present study, exposure to anti-anxiety drugs had an interaction with gender and was significantly associated with idiopathic CSCR among males only. Psychological stress and Type A behavior pattern have been implicated as contributing factors to the development of CSCR [4]. Conrad et al disclosed that CSCR patients were more stressed because of inadequate coping strategies [22]. In another case-control study, an increased use of psychopharmacologic drugs was identified as the independent risk factor for CSCR development [8]. Stress stimuli may increase secretion of cortisol and epinephrine, and these stress responses are more evident in males than in females [23],[24]. Without interviews using structured questionnaires, the extent of psychological stress in the study subjects could not be directly evaluated in the present study. However, the frequent prescription of anti-anxiety drugs in the male patients may imply that they were more likely to have problems in adaptation to psychological stress than the controls.

Except for exposure to anti-anxiety drugs with a modest OR in men, no other significant association between the reported risk factors, such as hypertension and pregnancy [8],[9], and idiopathic CSCR development was found in our study. Some possible explanations include that our study was large-scaled and population-based, and limited to the incident cases. Both Tittl et al’ s study (230 cases and 230 controls) and Haimovici et al’ s study (312 cases and 312 controls) were hospital-based and did not exclude the patients with recurrent or chronic CSCR [8],[9]. Similarly, the population-based study of Kitzmann et al., excluding patients with evidence of chronic CSCR, found no statistically significant risk factors for CSCR [18].

There are several strengths to our study. We retrospectively collected data from the NHIRD, which provided us with nationwide, population-based, and randomized claims information of insured people in Taiwan and, therefore, limited the selection bias. NHIRD contains every claims data that were recorded electronically, ensuring the accuracy and avoiding recall bias.

Several limitations to our study should be taken into consideration. First, diagnoses of CSCR and other comorbidities that are based on ICD-9 codes may be less accurate than those obtained individually through standardized procedures. Nevertheless, similar analysis to identify macular diseases has been used and proven valid in previous ophthalmic studies using the same dataset [25]–[29]. Besides, this limitation may be not significant in our study as ICD-9 code 362.41 is quite specific, and the diagnosis of new onset CSCR with the typical serous neurosensory retinal detachment is relatively straightforward. The atypical presentations observed in incident cases are more of a challenge and can mimic the clinical manifestations of the myriad macular diseases, which could have had atypical cases receive the diagnoses of other maculopathies. For lack of direct angiographic results, we excluded these cases to minimize the chance of a misclassification. However, the CSCR patients who were initially misdiagnosed with other maculopathies may also have been excluded and the incidence may therefore be underestimated. Second, personal information which might contribute to CSCR, such as Type A behavior pattern, psychological stress and drinking habit, were not available in the administrative database, and this could have compromised our results. Third, CSCR may be asymptomatic if the fovea is not involved. Subjects with eccentric CSCR or very mild symptoms may have been undiagnosed, which could have led to underestimation of incidence. Besides, these undetected patients may not seek ophthalmic care and could have been misclassified into the control group. To reduce the likelihood of this bias in the case-control study, we excluded potential controls who had never sought ambulatory or inpatient care provided by ophthalmologists during an 8-year period from 2000 to 2007.

### Conclusions

The present study provides large-scale, population-based evidence to estimate the incidence of idiopathic CSCR among adults in Taiwan. We found that the mean annual incidence of idiopathic CSCR among males was around 1.74-fold higher than that among females, and that exposure to anti-anxiety drugs was significantly associated with idiopathic CSCR among males. The difference in epidemiology of idiopathic CSCR between genders is still not well understood and further studies are warranted.

## References

[1] Wang M, Munch IC, Hasler PW, Prunte C, Larsen M (2008) Central serous chorioretinopathy. Acta Ophthalmologica 86: 126–145. 17662099 10.1111/j.1600-0420.2007.00889.x

[2] Gemenetzi M, De Salvo G, Lotery AJ (2010) Central serous chorioretinopathy: an update on pathogenesis and treatment. Eye 24: 1743–1756. 20930852 10.1038/eye.2010.130

[3] Ross A, Ross AH, Mohamed Q (2011) Review and update of central serous chorioretinopathy. Curr Opin Ophthalmol 22: 166–173. 21427570 10.1097/ICU.0b013e3283459826

[4] Yannuzzi LA (1987) Type A behavior and central serous chorioretinopathy. Retina 7: 111–131. 3306853 10.1097/00006982-198700720-00009

[5] Gelber GS, Schatz H (1987) Loss of vision due to central serous chorioretinopathy following psychological stress. Am J Psychiatry 144: 46–50. 3799839 10.1176/ajp.144.1.46

[6] Bouzas EA, Scott MH, Mastorakos G, Chrousos GP, Kaiser-Kupfer MI (1993) Central serous chorioretinopathy in endogenous hypercortisolism. Arch Ophthalmol 111: 1229–1233. 8363466 10.1001/archopht.1993.01090090081024

[7] Wakakura M, Ishikawa S (1984) Central serous chorioretinopathy complicating systemic corticosteroid treatment. Br J Ophthalmol 68: 329–331. 6712911 10.1136/bjo.68.5.329 PMC1040333

[8] Tittl MK, Spaide RF, Wong D, Pilotto E, Yannuzzi LA, et al (1999) Systemic findings associated with central serous chorioretinopathy. Am J Ophthalmol 128: 63–68. 10482095 10.1016/s0002-9394(99)00075-6

[9] Haimovici R, Koh S, Gagnon DR, Lehrfeld T, Wellik S, et al (2004) Risk factors for central serous chorioretinopathy: a case-control study. Ophthalmology 111: 244–249. 15019370 10.1016/j.ophtha.2003.09.024

[10] Bouzas EA, Karadimas P, Pournaras CJ (2002) Central serous chorioretinopathy and glucocorticoids. Surv Ophthalmol 47: 431–448. 12431693 10.1016/s0039-6257(02)00338-7

[11] Wakakura M, Song E, Ishikawa S (1997) Corticosteroid-induced central serous chorioretinopathy. Jpn J Ophthalmol 41: 180–185. 9243315 10.1016/s0021-5155(97)00027-0

[12] Carvalho-Recchia CA, Yannuzzi LA, Negrao S, Spaide RF, Freund KB, et al (2002) Corticosteroids and central serous chorioretinopathy. Ophthalmology 109: 1834–1837. 12359603 10.1016/s0161-6420(02)01117-x

[13] Karadimas P, Bouzas EA (2004) Glucocorticoid use represents a risk factor for central serous chorioretinopathy: a prospective, case-control study. Graefes Arch Clin Exp Ophthalmol 242: 800–802. 14986014 10.1007/s00417-004-0885-z

[14] Quillen DA, Gass DM, Brod RD, Gardner TW, Blankenship GW, et al (1996) Central serous chorioretinopathy in women. Ophthalmology 103: 72–79. 8628563 10.1016/s0161-6420(96)30730-6

[15] Spaide RF, Campeas L, Haas A, Yannuzzi LA, Fisher YL, et al (1996) Central serous chorioretinopathy in younger and older adults. Ophthalmology 103: 2070–2079. 9003341 10.1016/s0161-6420(96)30386-2

[16] Fukunaga K (1969) Central chorioretinopathy with disharmony of the autonomous nerve system. Nihon Ganka Gakkai Zasshi 73: 1468–1477. 5390583

[17] Desai UR, Alhalel AA, Campen TJ, Schiffman RM, Edwards PA, et al (2003) Central serous chorioretinopathy in African Americans. J Natl Med Assoc 95: 553–559. 12911253 PMC2594640

[18] Kitzmann AS, Pulido JS, Diehl NN, Hodge DO, Burke JP (2008) The incidence of central serous chorioretinopathy in Olmsted County, Minnesota, 1980–2002. Ophthalmology 115: 169–173. 18166410 10.1016/j.ophtha.2007.02.032

[19] National Health Insurance Research Database. Available at: http://nhird.nhri.org.tw/en/Research.html. Accessed 18 December 2012.

[20] Leveque TK, Yu L, Musch DC, Chervin RD, Zacks DN (2007) Central serous chorioretinopathy and risk for obstructive sleep apnea. Sleep Breath 11: 253–257. 17457629 10.1007/s11325-007-0112-3

[21] Chan WM, Lai TY, Tano Y, Liu DT, Li KK, et al (2006) Photodynamic therapy in macular diseases of asian populations: when East meets West. Jpn J Ophthalmol 50: 161–169. 16604394 10.1007/s10384-005-0259-z

[22] Conrad R, Bodeewes I, Schilling G, Geiser F, Imbierowicz K, et al (2000) Chorioretinopathia centralis serosa und psychische Belastung. Ophthalmologe 97: 527–531. 10994328 10.1007/s003470070059

[23] Kirschbaum C, Wust S, Hellhammer DH (1992) Consistent sex differences in cortisol responses to psychological stress. Psychosom Med 54: 648–657. 1454958 10.1097/00006842-199211000-00004

[24] Harbin TJ (1989) The relationship between the type A behavior pattern and physiological responsivity: a quantitative review. Psychophysiology 26: 110–119. 2922451 10.1111/j.1469-8986.1989.tb03138.x

[25] Hu CC, Ho JD, Lin HC (2009) Retinal vein occlusion and the risk of acute myocardial infarction: a 3-year follow-up study. Br J Ophthalmol 93: 717–720. 19208680 10.1136/bjo.2008.151605

[26] Hu CC, Ho JD, Lin HC (2010) Neovascular age-related macular degeneration and the risk of stroke: a 5-year population-based follow-up study. Stroke 41: 613–617. 20150546 10.1161/STROKEAHA.109.571000

[27] Chou KT, Huang CC, Tsai DC, Chen YM, Perng DW, et al (2012) Sleep Apnea and Risk of Retinal Vein Occlusion: A Nationwide Population-Based Study of Taiwanese. Am J Ophthalmol 154: 200–205.e1. 22464364 10.1016/j.ajo.2012.01.011

[28] Tsai DC, Huang CC, Chen SJ, Chou P, Chung CM, et al (2012) Central serous chorioretinopathy and risk of ischaemic stroke: a population-based cohort study. Br J Ophthalmol 96: 1484–1488. 22976583 10.1136/bjophthalmol-2012-301810

[29] Tsai DC, Huang CC, Chen SJ, Chou P, Chung CM, et al.. (2012) Increased risk of erectile dysfunction among males with central serous chorioretinopathy - a retrospective cohort study. Acta Ophthalmol In press. 10.1111/j.1755-3768.2012.02528.x 22998678

